# On the use of preclinical imaging to explore the principles of brain function in rodent models and their relevance for illnesses of the human mind

**DOI:** 10.1038/s41398-022-01924-y

**Published:** 2022-04-11

**Authors:** Valerio Zerbi

**Affiliations:** 1 Neural Control of Movement Lab, Department of Health Sciences and Technology, ETH Zürich, Zürich, Switzerland; 2 Network Physiology Lab, School of Engineering (STI), Institute of Bioengineering (IBI), EPFL, Lausanne, Switzerland; 3 Center for BiomedicalImaging (CIBM), Lausanne, Switzerland

**Keywords:** Neuroscience, Psychiatric disorders, Scientific community

Dear Editor,

We recently published in Translational Psychiatry an article that examine the strategies for evaluating brain function at the whole-brain level [1]. In this review, we covered several methods, from functional MRI to functional ultrasound to calcium imaging. For each technique, we wrote a brief history of its development, the physical notion, some key applications, its potentials, and its limitations. We concluded that methods for imaging the rodent brain at the network level are growing and will advance our understanding of brain function.

A commentary by Zhuo and colleagues further enhances the complexity of addressing the issue of “translation” from animal models to patients for the discipline of psychiatry [2]. They propose that the approaches employed to develop an animal model for a psychiatric disease need to be thoroughly scrutinized and, perhaps, revised. For example, most rodent models of mental diseases are to-date established using a simple pharmacological infusion [3] and/or psychosocial stimulation [4]. The key concern posed, however, is how these manipulations change the brain’s structure and function, and whether these models genuinely reflect the pathophysiology of human mental illnesses. Especially since it is difficult to evaluate whether one can speak of inverse inference from rodents to humans.

This is a true and acceptable statement. However, this is exactly what preclinical imaging aims to deliver. By mapping the dynamic responses of brain networks in animal models and compare them, if possible, with those reported in clinical studies, we can obtain quantitative data and parameters to establish whether our models are effectively translational [5]. If these metrics demonstrate temporal and spatial similarity in network-level modifications as those observed in humans, we can pursue further inquiry utilizing more intrusive and more specific methods for brain recordings in animal models. Otherwise, we must have the confidence and the correctness to move forward and attempt other solutions.

Two recent examples. In 2019 we established a causal association between activity of the noradrenergic nucleus locus coeruleus (LC) and the engagement of numerous large-scale brain networks in mice, in particular of the salience and amygdala networks [6]. In addition, we could link network-changes with direct markers of norepinephrine (NE) turnover and with the distribution of NE receptors over the entire brain. The hypothesis that specific brain networks dynamics are related to LC activity and to NE receptor density derives from stress-research and pharmacological studies in humans [7, 8]. However, since it is impossible to selectively stimulate LC in people, it has remained a hypothesis for more than a decade. Our preclinical work helped confirm this causal relationship and this has direct implications for interpreting the results of clinical imaging studies on stress and anxiety behavior.

More recently, the Gozzi lab described how chronic local neuronal suppression via overexpression of a potassium channel or acute silencing via chemogenetics result in a paradoxical hyper-connectivity [9]; an intriguing finding often reported in humans after stroke [10] and in early stages of Alzheimer’s disease [11], but never truly understood. Using in vivo electrophysiology, they showed local inhibition improves low frequency (0.1–4 Hz) oscillatory power via suppression of neuronal activity not phase-locked to slow rhythms, resulting in increased slow and δ band coherence between areas that display fMRI overconnectivity. These data present causal evidence that cortical inactivation can counterintuitively augment fMRI connectivity via greater, less-localized slow oscillatory processes. Once again, this could be only achieved by combining functional MRI and electrophysiology with neuromodulation in animal models.

These and other examples give a peek of what the future of preclinical imaging might look like: a field of research capable of delivering causal explanations to the hypotheses presented by human neuroscience, neurology and psychiatry.

Lastly, I would argue against statements like “the computational complexity of human brains is *billions of times* that of mouse brain”. While this may be true from a numerical standpoint of mere neuronal counts, preclinical neuroimaging’s objective should not be per se to map every single neuron in real time but of identifying the general neural and cellular principles governing the assembly of brain networks and its breakdown in brain disorders. The field is relatively new but is moving fast and has already produced some important insights. The future is challenging and will require time, devotion and an optimal synergy between engineering, chemistry, biology, and computer science. If the community will be patient and supportive enough, there will be further important discoveries in the future.
